# Fecal microbiota transplantation in the treatment of irritable bowel syndrome: a single-center prospective study in Japan

**DOI:** 10.1186/s12876-022-02408-5

**Published:** 2022-07-14

**Authors:** Motonobu Hamazaki, Tsunaki Sawada, Takeshi Yamamura, Keiko Maeda, Yasuyuki Mizutani, Eri Ishikawa, Satoshi Furune, Kenta Yamamoto, Takuya Ishikawa, Naomi Kakushima, Kazuhiro Furukawa, Eizaburo Ohno, Takashi Honda, Hiroki Kawashima, Masatoshi Ishigami, Masanao Nakamura, Mitsuhiro Fujishiro

**Affiliations:** 1 Department of Gastroenterology and Hepatology, Nagoya University Graduate School of Medicine, 65 Tsurumai-cho, Showa-ku, Nagoya, Japan; 2 Department of Endoscopy, Nagoya University Hospital, 65 Tsurumai-cho, Showa-ku, Nagoya, Japan

**Keywords:** Fecal microbiota transplantation, Irritable bowel syndrome, Microbiome, Gastrointestinal endoscopy

## Abstract

**Background:**

Fecal microbiota transplantation (FMT) is a potential treatment for irritable bowel syndrome (IBS), but its efficacy in Japanese IBS patients is unknown. This study aimed to evaluate the efficacy, side effects, and microbiome changes following FMT in Japanese IBS patients.

**Methods:**

Seventeen Japanese patients with refractory IBS received FMT (4 donors) under colonoscopy. Responders were defined by an improvement in the IBS severity index (IBS-SI) of 50 points or more after 12 weeks. We evaluated the IBS-SI and Bristol Stool Form Scale (BSFS) and compared the diversity and microbiome before and 12 weeks after FMT. For the microbiome, we analyzed the V3–V4 region of the 16S rRNA gene.

**Results:**

IBS-SI decreased an average of 115.58 points after 12 weeks, and 10 patients (58.8%) were considered responders. Eight patients with diarrhea (66.7%) and three patients with constipation (60.0%) showed improvement in the BSFS. Two patients complained of mild abdominal pain, but there were no cases with severe side-effects. α-diversity was increased only in the responder group (*p* = 0.017). Patients who closely paralleled the donor microbiome had a higher rate of IBS-SI improvement. The relative abundance of *Neisseria* and *Akkermansia* increased and *Desulfovibrio* and *Delftia* were decreased in the responder group after FMT.

**Conclusions:**

Following FMT, about 60% of Japanese patients with IBS showed improvement in both the IBS-SI and BSFS, without severe side effects. Increased α-diversity and similarity to the donor microbiome after FMT may be associated with better treatment effects.

*Trial registration:* This study was registered in the University Hospital Medical Information Network Clinical Trial Registration (UMIN000026363). Registered 31 May 2017, https://rctportal.niph.go.jp/s/detail/um?trial_id=UMIN000026363. The study was registered prospectively.

**Supplementary Information:**

The online version contains supplementary material available at 10.1186/s12876-022-02408-5.

## Background

Worldwide, the prevalence of irritable bowel syndrome (IBS) is 11.2% [1], while in Japan, it is slightly higher at 13.5% [2]. Although IBS is not a fatal disease, patients’ quality of life (QOL) is significantly impaired because behavioral restrictions interfere with social activities [3–5]. Additionally, the economic loss due to this condition is significant [6]. However, current IBS treatments are limited, and some patients do not improve with existing treatments [7–9], mainly because the etiology of IBS is multifactorial and not completely understood. It has been suggested that several factors play decisive roles in its pathophysiology, including genetics, social learning, diet, the intestinal microbiome, low-grade chronic intestinal inflammation, and abnormal gastrointestinal endocrine cells [1]. Recently, there have been many reports regarding the relationship of those factors with the gut microbiome in IBS patients [10–15]. These studies have indicated that the microbiome composition in IBS patients differs (i.e., dysbiosis) from that in healthy people and may be associated with IBS etiology [11–15]. Current research has also indicated that dysbiosis of the microbiome is associated with abnormalities in the gastrointestinal and brain-gut axis functions of IBS patients [16–18]. Thus, a novel treatment to reverse dysbiosis is needed. Manipulation of the microbiome in IBS patients through prebiotics, probiotics, and synbiotics has been reported, but sufficient effects have not been obtained [10, 19, 20]. FMT has been proven to be highly effective in refractory *Clostridium difficile* infectious enteritis [21] and was expected to show promising results for IBS. However, so far, the effect of FMT for IBS has been controversial [22–28]. While some previous RCTs regarding FMT in patients with IBS reported a positive effect [24–27], others did not [22, 23, 28]. Variations in FMT protocols, including donor selection criteria, stool volume used, and method of administration, were noted as reasons for these conflicting results [22–28]. In addition, because the microbiome varies depending on race/ethnicity and lifestyle [29], the results may differ depending on the target patient population.

Even in the Japanese population, there is only one report of FMT for IBS [30]; in order to establish more effective FMT protocol in Japan, studies analyzing the microbiome changes in Japanese IBS patients are necessary. Therefore, this study aimed to clarify the efficacy, side effects, and microbiome changes in Japanese IBS patients following FMT using our protocol.

## Methods

### Study design

In this single-center prospective study, the IBS severity index (IBS-SI) and the Bristol stool form scale (BSFS) were used to evaluate the effects of FMT in patients with refractory IBS. In addition, we evaluated the change in diversity and differences in the microbiome before and at 12 weeks after FMT using the linear discriminant analysis effect size (LEfSe).

This study was registered in the University Hospital Medical Information Network Clinical Trial Registration (UMIN000026363). All clinical and stool sample information was anonymized, and a database was constructed.

### Selection of patients and donors

Among IBS patients diagnosed according to Rome IV criteria [31] from December 2018 to December 2019, men and women aged 20 years or older who were refractory to existing treatment with medication for more than one year were included in this study.

The exclusion criteria were as follows: (1) consent was not obtained to participate in the study, (2) pregnancy, and (3) the attending physician determined that the patient was not appropriate for this study. Reasons for (3) were (a) change of treatment for IBS in 12 weeks, (b) patients used antibiotics, (c) receiving treatment for a serious illness, (d) difficulty in performing lower endoscopy, and (e) FMT follow-up was interrupted within six months. As a result, a total of 21 patients were enrolled and received FMT. However, four patients dropped out and the final evaluation included 17 patients. Of the four patients who withdrew from the study, two patients used antibiotics within three months after FMT, and two patients discontinued follow-up due to inconvenience after FMT.

The FMT protocol was developed based on a previously published report [32]. To determine donor criteria, blood tests, stool tests, and interviews were conducted for men and women over the age of 20 who did not have any underlying disease, as shown in Additional file 1: Table S1.

When the recipient requested that a relative served as the donor, their relative was selected as the first candidate for the donor; however, when there was no qualified individual, one of the four donors was randomly selected. Donor feces were stored using AneroPack® (MITSUBISHI GAS CHEMICAL Company, Inc. Tokyo, Japan) immediately after defecation. Feces were purified within 3 h of defecation. The stool sample was purified by suspending the donor stool in saline and removing the excess residue through a metal mesh and gauze filter. Purification was adjusted to a consistency that did not block the spray tube used during FMT. A single dose comprised about 30 g of donor stool dissolved in 100 mL of physiological saline to form a stool suspension purified as described above and stored frozen at − 80 °C until the day of FMT. For FMT, 100 mL of frozen fecal suspension was thawed in warm water at 37 °C for approximately 2 h on the day of FMT. Under sedation, a lower gastrointestinal endoscope was used to transplant the fecal suspension into the right colon using a spray tube. Then, 50 ml of the fecal suspension was applied to the ileocecal region and 50 ml to the hepatic flexure side from the ascending colon to the transverse colon. After spraying the entire amount, the endoscope was quickly removed to prevent suction. Upon completion, the patient was instructed to rest in the right lateral decubitus position for 2 h.

### Clinical outcomes

The IBS-SI and BSFS were recorded before, and four, eight, and 12 weeks after FMT to assess its therapeutic efficacy. The IBS-SI has a total of 500 points, with 100 points each for the following five items: abdominal pain (severity), abdominal pain (frequency), bloating (severity), satisfaction with bowel movements, and disability for daily activities of living (> 75, remission; 75–175, mild IBS; 176–300, moderate IBS; > 300, severe IBS [33]. In this study, the group in which IBS-SI decreased by 50 points or more at 12 weeks as compared with before FMT was defined as the responder group [32, 34]. The remaining patients were defined as the non-responder group.

The transition of each time point of BSFS was evaluated, and improvement cases were defined as patients with a mean value of BSFS per week that improved from a score of 3 to 5, at 12 weeks after FMT [33].

### Safety assessment

Evaluation of adverse events was performed six months after FMT. Abdominal pain, fever, nausea, and abdominal bloating were evaluated as adverse events. Severity was classified as follows: mild, no intervention required; moderate, outpatient intervention; and severe, inpatient treatment required. When symptoms appeared, the cause was diagnosed using blood tests and computed tomography (CT). The relationship between the cause of the adverse event and FMT was evaluated by two or more gastroenterologists.

### Analysis of microbiome

The microbiome analysis was performed in a similar manner as previously reported [35]. In summary, DNA purification was performed using the DNeasy PowerSoil Kit (Qiagen, Hilden, Germany). Sequencing was performed using Miseq (Illumina, San Diego, CA, USA) using the universal primers (forward: 5-TCGTCGGCAGCGTCAGATGTGTATAAGAGACAGCCTACGGGNGGCWGCAG-3 and reverse: 5-GTCTCGTGGGCTCGGAGATGTGTATAAGAGACAGGACTACHVGGGTATCTAATCC-3) targeting the bacterial 16S rRNA V3–4 region.

QIIME 1.9.1 software [36] was used to bin the sequences based on their barcode and to trim the primers and barcodes. Quality check was performed using split_libraries_fastq.py and the average input sequences were 71,995.28 and the filtered total number of sequences was 71,785.18. In addition, USEACH 6.1 software [37] was used to remove chimeric sequences. Operational taxonomic unit formation was performed using QIIME reference optimal picking at 97% similarity [38]. We used full Greengenes version 13_8 [39] as an open reference database. MicrobiomeAnalyst [40] was used for microbiome analysis and for diversity comparison and LEfSe [41] was used for comparison between the two groups. Both software packages were used in their default settings. Principal Coordinate Analysis (PCoA) plots (Distance method: Bray–Curtis dissimilarity) were also created in MicrobiomeAnalyst.

**Fig. 1:**
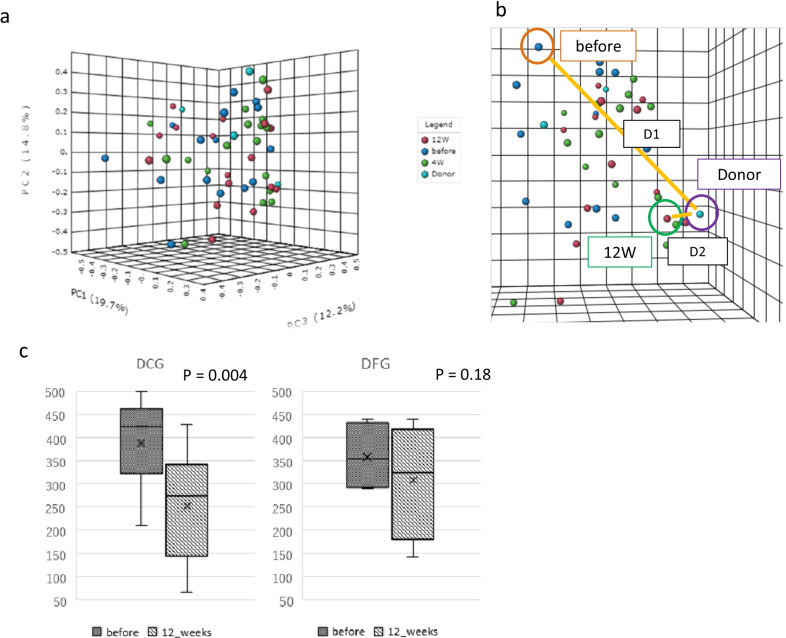
Principal coordinate analysis plot in donor and irritable bowel syndrome patients after fecal microbiota transplantation. **a** Principal Coordinate Analysis (PCoA) plot of patients (N = 17) with irritable bowel syndrome (IBS) and donors (N = 4). **a**, **b** Changes in patients’ microbiome were evaluated based on the distance traveled on the PCoA plot, as defined below. **c** Change of IBS severity index (IBS-SI) in patients classified as belonging to the Near Donnor Group (NCG, N = 13) and the Far Donor Group (FDG, N = 4) before and at 12 weeks after fecal microbiota transplantation (FMT)

Furthermore, based on PCoA plot, we examined whether changes in a patient’s microbiome made it resemble their donor’s microbiome after FMT. Specifically, PCoA plot was calculated using MicrobiomeAnalyst (Fig. 1a). The distance between the donor and the pre-FMT patient before FMT was set as D1, and the distance between the donor and the post-FMT 12 weeks later patient was set as D2, and they were calculated three-dimensionally from the coordinate axes (Fig. 1b). This defined D1–D2 > 0: Near Donor Group (NDG: approaching group) and D1–D2 < 0: Far Donor Group (FDG: moving away group).

### Statistical analysis

Data were analyzed using SPSS (IBM SPSS Statistics version 26). A *p*-value < 0.05 was considered to indicate significance. The two groups were compared using the Wilcoxon rank-sum test for distributed continuous variables. When there were more than three groups, they were compared using the same statistical method, Wilcoxon, between each group. Continuous data are represented as the mean ± standard deviation and analyzed by a paired *t*-test. The LEfSe method was used to analyze the changes in the microbiome.

## Results

### Patient background and characteristics

Eleven men and six women with IBS were included in this study, with a median age of 54 years, mean body mass index of 21.8 kg/m^2^, and a median disease duration of 6.9 years. The types of IBS were IBS-D (diarrhea) in 12 cases and IBS-C (constipation) in five cases, and the average value of IBS-SI before FMT was 381.2 ± 81.9. The median age of the four donors (A to D) was 31 years (range, 22–38 years), including two men and two women (Table 1). The number of patients per donor was nine for Donor A, two for Donor B, and three for Donors C and D.

**Table 1:** Baseline characteristics of patients and donors

Patients	(N = 17)
Age (years), median (range)	54 (20–62)
Sex ratio M/F (n)	11/6
Body mass index (kg/m^2^), median (range)	21.8 (15–32)
Disease duration (years), median (range)	6.9 (1–40)
IBS-SI (range)	407 (210–500)
IBS-type (n, %)	
IBS-D	12 (70.6%)
IBS-C	5 (29.4%)

Donors	(N = 4)
Age (years), median (range)	31 (22–38)
Sex ratio M/F (n)	2/2

*IBS* Irritable bowel syndrome, *IBS-D* diarrhea-predominant IBS, *IBS-C* constipation-predominant IBS, *M* male, *F* female

### Therapeutic effects of FMT

#### IBS-SI and BSFS

**Fig. 2:**
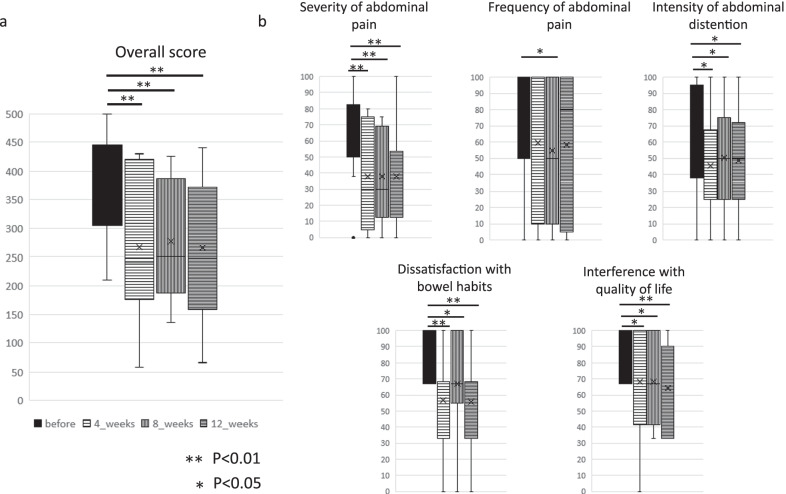
Irritable bowel syndrome severity index. **a** Overall score. **b** Subscale scores for five items of irritable bowel syndrome severity index (IBS-SI) in patients treated with fecal microbiota transplantation (FMT)

The median values (range) of IBS-SI were 407 (210–500) before FMT, and 240 (58–430) at 4 weeks, 250 (136–426) at 8 weeks, and 289 (66–440) at 12 weeks after FMT. Comparing pre-FMT and IBS-SI, a tendency for significant improvement was observed in all periods of 4, 8, and 12 weeks (*p* = 0.003, 0.001, and 0.001, respectively) (Fig. 2a).

**Fig. 3:**
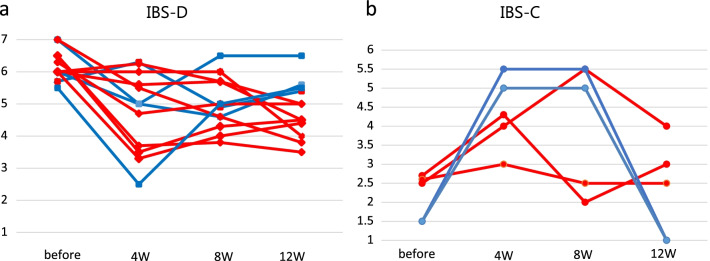
Bristol stool form scale. **a** Change in stool form scale in responder (red line) and non-responder (blue line) patients with diarrhea-predominant irritable bowel syndrome (IBS-D, N = 12) and **b** constipation-predominant irritable bowel syndrome (IBS-C, N = 5) using the Bristol stool form scale before and 4, 8, and 12 weeks after fecal microbiota transplantation (FMT)

Ten (58.8%) patients were responders. A significant improvement was observed at four, eight, and 12 weeks for items other than abdominal pain frequency (*p* < 0.05). The frequency of abdominal pain was significantly different only at 8 weeks as compared with before FMT. The effective rate for each donor was 66.7% (6/9) for donor A, 100% (2/2) for donor B, 33.3% (1/3) for donor C, and 33.3% (1/3) for donor D. According to sex, effective rate was 54.5% (6/11) for men and 66.7% (4/6) for women. The relative abundance of four donor microbiome at the phylum and genus level is shown in Additional file 2: Fig. S1. An improvement of BSFS was observed in eight patients (66.7%) of IBS-D and three patients (60.0%) of IBS-C at 12 weeks after FMT (Fig. 3).

#### Adverse events

Two patients experienced abdominal distension after FMT. One case lasted about 12 h after FMT treatment, and the other lasted for two days after FMT treatment. These events were considered to be related to FMT because they occurred after FMT without any other cause. The cases were classified as mild because there were no problems in daily life; there were no abnormal findings on CT or blood tests, and both cases improved in a short period of time without therapeutic interventions.

### Changes in the microbiome

#### Changes in α-diversity and β-diversity before and after FMT

**Fig. 4:**
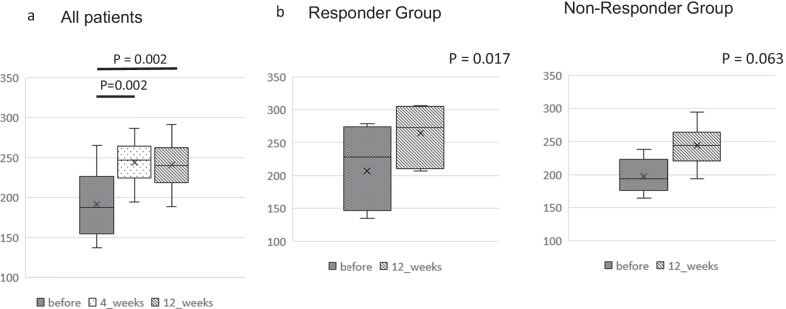
Alpha-diversity of gut microbiome in patients with irritable bowel syndrome after fecal microbiota transplantation. **a** Left: Diversity (Chao1) of microbiome in 17 patients with irritable bowel syndrome (IBS) before and at 4 and 12 weeks after fecal microbiota transplantation (FMT). **b** Right: Diversity (Chao1) of microbiome in 10 patients in responders (N = 10) and non-responders (N = 7) before and at 12 weeks after fecal microbiota transplantation (FMT)

The median α-diversity index (Chao1 index) for all IBS patients was 187.5. It was 246.8 (*p* = 0.002) at 4 weeks, and 239.9 (*p* = 0.002) at 12 weeks after FMT, showing a significant increase from before FMT at both time points (Fig. 4a). In addition, when comparing before and after 12 weeks, α-diversity increased in the responder group (ten subjects, *p* = 0.017, Fig. 4b), and no significant increase in α-diversity was observed in the non-responder group (seven subjects, *p* = 0.063, Fig. 4c).

The PCoA plots of patients and donors are shown in Fig. 1a. Based on the PCoA plot, we evaluated whether changes in a patient’s microbiome resembled their donor’s microbiome after FMT. Figure 4b schematically shows D1 and D2. The PCoA plot displays closer to the microbiome. In other words, when comparing D1 and D2, it is possible that D2 is more similar to the donor’s microbiome. Using this methodology, we defined D1 > D2 on the PCoA plot (that is, the microbiome after FMT is more similar to the donor’s microbiome) as the Near Donor Group (NDG) (Fig. 1b). Patients with D1 < D2 were defined as the Far Donor Group (FDG). Among the ten patients in the responder group, nine belonged to the NDG and one to the FDG. Furthermore, among the seven patients in the non-responder group, four belonged to the NDG and three to the FDG. Before and after 12 weeks of FMT, the NDG patients had significantly improved IBS-SI, while FDG patients showed no significant improvement.

#### Comparison of microbiome before and after FMT

**Fig. 5:**
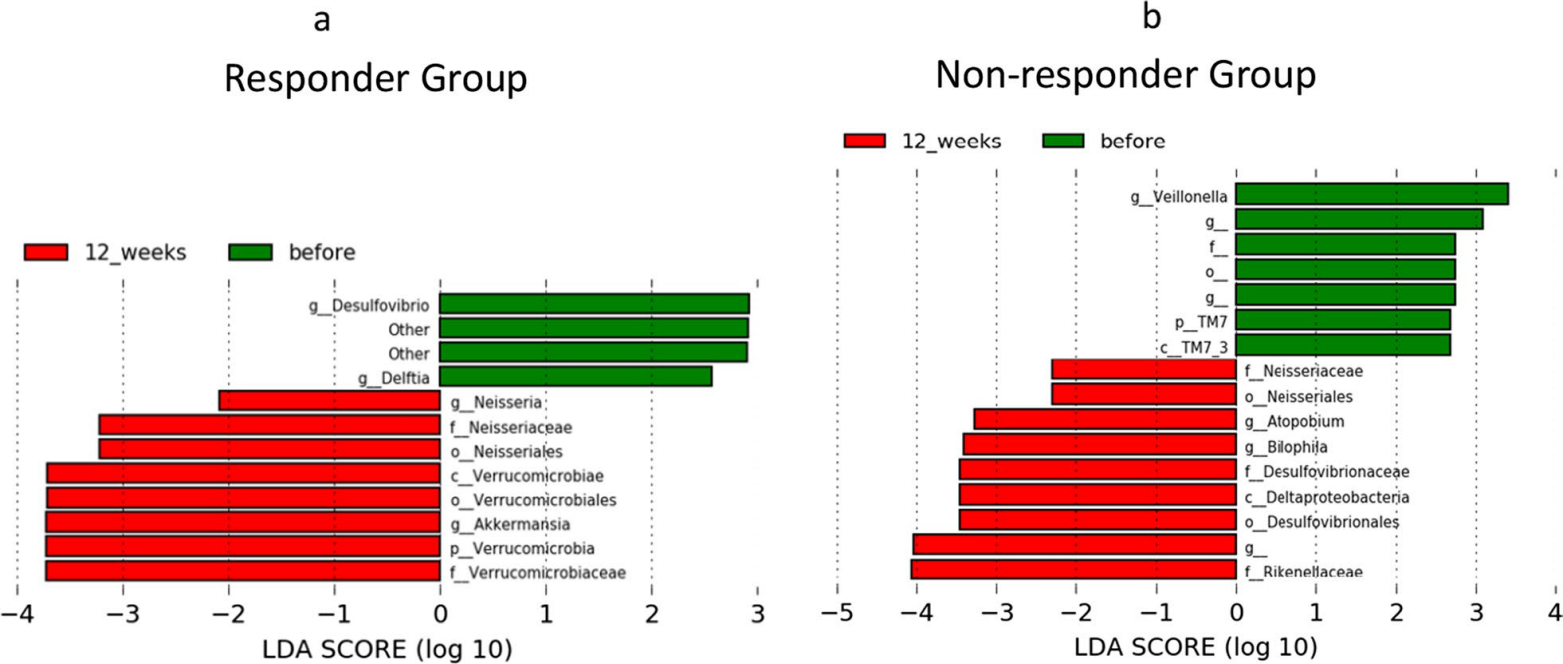
Differences in microbiome before and at 12 weeks after fecal microbiota transplantation. Significant differences were found in the microbiome before fecal microbiota transplantation (FMT) and at 12 weeks by linear discriminant analysis effect size (LEfSe) in the responder (N = 10) and non-responder (N = 7) groups. **a** In the responder group, two genera, Neissria and Akkermansia, were significantly increased, and two genera, Desulfovibrio and Delftia, were significantly decreased. **b** In the non-responder group, two genera, Atopobium and Bilophila, increased and one genera, Veillonella, decreased

To evaluate changes in the microbiome, fecal bacteria were compared before and at 12 weeks after FMT between the responder and non-responder groups (Fig. 5). The proportion of the microbiome is shown in Additional file 3: Fig. S2, and fecal bacteria were compared before and after FMT in all patients is shown in Additional file 4: Fig. S3. A heat map comparing the whole group is also shown in Additional file 5: Fig. S4. Each heat map was sorted by some factors, including timing (online resource, Additional file 5: Fig. S4a), effectivity (online resource, Additional file 5: Fig. S4b), and IBS-type (Additional file 5: Fig. 4c).

In the responder group, the genera *Neisseria* and *Akkermansia* significantly increased, and the genera *Desulfovibrio* and *Delftia* significantly decreased after 12 weeks. In the non-responder group, the bacterial genera *Atopobium* and *Veillonella* decreased after 12 weeks (Fig. 5). This trend indicates that changes in the microbiome occurred in both the responder and non-responder groups. However, there were differences in the changes of intestinal bacteria, and the effect of FMT is indicated by the type of bacterial changes.

## Discussion

Because refractory IBS negatively impacts patient’s QOL, new IBS treatments are desired. In this study, we evaluated the efficacy of FMT by lower gastrointestinal endoscopic administration of 30 g of frozen stool, and the effective rate was 58.8%.

Kanchana et al. conducted the latest meta-analysis of FMT for IBS [42]. In their single-arm study using upper or lower gastrointestinal endoscopy, the efficacy of FMT was determined to be 59.5%. Notably, the efficacy (58.8%) in the current study, also a single-arm study, was similar to this previous report. However, few previous studies have reported on the placebo effect (43–44%) of bowel cleansing before lower gastrointestinal endoscopy on IBS symptoms [43, 44]. Hence, possibility of placebo effect on the results of this study cannot be ruled out. Some studies also reported that women are more likely to respond to FMT than men [26, 45]. In our study, sex based efficacy was 54.5% (6/11) for men and 66.7% (4/6) for women. This suggests that if the male/female ratio in this study had been equal, the efficacy would have been a little higher. In order to resolve these issues in the future, we consider that studies comparing the results of these studies with those of placebo based studies, with a uniform set of background conditions are needed.

Regarding FMT protocol in terms of treatment efficacy, a meta-analysis using randomized controlled trials did not find usefulness in the IBS-SI as compared to the control group [42]. Aroniadis et al. performed FMT using capsules as a double-blind, randomized, placebo-controlled trial, but did not show the effectiveness of IBS-SI [22]. Similarly, FMT using Sofie Ingdam Halkjæ capsules was performed in another study; while the IBS-SI in the FMT group improved, the placebo group improved even more; hence, efficacy could not be determined [23]. On the other hand, FMT using nasojejunal, upper gastrointestinal endoscopy, and lower gastrointestinal endoscopy was found to be more efficacious than placebo in previous RCTs [24–27]. The efficacy in the present study was 58.8%, while the efficacy of FMT for IBS patients in previous RCTs have been reported as 89.1% using upper gastrointestinal [24] endoscopy, 56% using nasojejunal [26], and 65% using lower gastrointestinal endoscopy　[25]. Due to differences in protocols other than the method of administration, it is not possible to make a general comparison, and at present it is not known whether FMT into the upper or lower gastrointestinal tract is more effective. Furthermore, we consider that safety and patient acceptability are important factors in the choice of administration method. Recent studies have shown that the dose of fecal transplantation is important for the efficacy of FMT [24]. El-Salhy et al. reported that 76.9% and 89.1% of patients who received 30 g and 60 g FMT, respectively, elicited a response [24]. The study reported the best treatment efficacy (89.1%) was achieved using upper gastrointestinal endoscopy with 60 g stool, but there is no report to show whether increasing the dose of stool in large intestine administration increases the efficacy. For instance, Johsen et al. showed 65% efficacy with 50–80 g [25], which was better than our results.

Regarding the safety of FMT, only mild adverse events occurred in 11.8% (2/17) of patients. Side effects of IBS may also include those associated with lower gastrointestinal endoscopy. Wang et al. summarized the complications associated with FMT [46]. According to their report, the total incidence of adverse events was 28.5%. They also reported that the incidence of adverse events through the upper gastrointestinal route (by nasoenteric tube or upper gastrointestinal endoscopy) was higher than that through the lower gastrointestinal route (by enema or lower gastrointestinal endoscopy) (43.9% vs. 20.6%). In our study using lower gastrointestinal endoscopy, the safety of FMT was superior to that of these data.

Thus, we believe that the use of lower gastrointestinal endoscopy may be a superior method in terms of efficacy and safety, although we need to determine the optimal protocol through future studies.

We investigated the relationship between the therapeutic effect of FMT and changes in the microbiome. Changes in the microbiome were evaluated for α-diversity, β-diversity, and the differences between each bacterium. α-diversity indicates the diversity of a single sample and is an indicator of the diversity of an individual’s microbiome. Similar to previous reports [23, 30], our study showed that the improvement of α-diversity in patients after FMT might be involved in the therapeutic effect. It has been reported that a high α-diversity is important for donor conditions with a high FMT effect, the supposed “super-donor” [47]. Accumulating cases with FMT limited to donor A, who had the highest α-diversity in the current study, may enhance the treatment effect. The β-diversity is an index showing the degree of difference in sample diversity; the greater the distance, the greater the difference in the composition of the two microbiomes. Some studies of *C. difficile* revealed that after FMT, the PCoA plot of patients more closely resembled that of donors [48, 49]. Halkjaer et al. reported that changes in the PCoA plot showed that the microbiome of patients with IBS looked more similar to that of the donor after FMT and were clearly different from those in a placebo group [39]. However, its association with clinical symptoms has not been fully investigated [39]. In our study, the PCoA plot included patients who approximated the donor (NDG) and those who did not (FDG). The IBS-SI significantly improved in the NDG group, but not in the FDG group (Fig. 4c). Although such studies have not been reported to date, it has been suggested that the proximity to the microbiome of the donor may be a factor in the therapeutic effect.

Moreover, we observed some changes in the microbiome before and after FMT. At the genus level, *Akkermansia* and *Neisseria* showed a significant increase in the responder group. Of these, *Akkermansia* is more abundant in healthy individuals than in patients with bowel diseases [50]. *Akkermansia* is reported to have an anti-inflammatory effect that enhances the tight junction between intestinal epithelial cells and the barrier function in the mucous layer to reduce the permeability of the intestinal mucosa [51]; in addition, it has anti-metabolic syndrome and anti-diabetic effects [52–54]. An FMT study also reported an inverse correlation between the proportion of *Akkermansia* and the reduction of abdominal pain in patients with IBS [55], which may have contributed to the therapeutic effect in this study. At the genus level, *Desulfovibrio,* which are sulfate-reducing bacteria that produce hydrogen sulfide using hydrogen, decreased significantly only in the responder group. There are reports that hydrogen sulfide may affect intestinal motility and visceral hypersensitivity [56–58], and that IBS-C and sulfate-reducing bacteria are also involved [56]. Therefore, we hypothesize that the increase or decrease of these bacterial genera may be related to the treatment effect. According to a study conducted by Mizuno et al. in Japan [30], the treatment response was high in patients who received donor stools rich in *Bifidobacterium*. Similarly, in our study of Japanese patients, the therapeutic effect of FMT may have been affected by the altered bacteria on the patient side. Interestingly, *Akkermansia* and *Neisseria*, which were significantly increased in the responder group in our study, were almost absent in the donors’ stool. This phenomenon, reported by Hong et al. [59], can be considered as an increase in specific bacteria suitable for that environment after a dramatic change in the intestinal environment caused by FMT. In other words, the mechanism by which FMT exerts its therapeutic effect is not only the accumulation of the administered bacteria but also the transformation of the environment to one in which specific good bacteria are able to grow.

There were several limitations to our study. This study performed only a single group analysis with a small number of cases, and it was not a randomized control trial. Therefore, the change in IBS-SI due to the placebo effect has not been evaluated. In addition, there was a bias in the number of times the donor sample was used. Future one-donor studies using donors with the highest α-diversity are needed to rule out donor side factors. In addition, IBS has not been examined for each type. In terms of the immunological profile of IBS, no significant differences were found between IBS-D and IBS-C [42, 60]. However, the microbiome may differ depending on the defecation status, so it will be necessary to consider each status in the future.

## Conclusions

In this study, FMT in Japanese patients with IBS was performed safely, and a therapeutic effect was observed in 58.8% of patients. The effect of FMT involves changes in the microbiome, including its diversity. In addition, the therapeutic effect could be related to an increase in α-diversity, the proximity to the microbiome of healthy donors, and the increase or decrease of certain bacterial genera. FMT is expected to be a promising treatment for refractory IBS, and this study will contribute to the improvement of therapeutic efficacy of FMT.

## Supplementary Information

**Additional file 1: Table S1.** Screening Test and Interview of Donors.

**Additional file 2: Fig. S1.** The relative abundance of all donors’ microbiome at the phylum and genus level.

**Additional file 3: Fig. S2.** The relative abundance of all patients’ microbiome at the phylum and genus level.

**Additional file 4: Fig. S3.** Differences in all patients’ microbiome before and after Fecal Microbiota Transplantation.

**Additional file 5: Fig. S4.** Each heat map was sorted by timing, effectivity, and IBS-type.

## Data Availability

The datasets used and/or analysed during the current study are available from the corresponding author on reasonable request.

## Abbreviations

BSFS: Bristol stool form scale
FDG: Far Donor Group
FMT: Fecal microbiota transplantation
IBS: Irritable bowel syndrome
IBS-C: Constipation-predominant irritable bowel syndrome
IBS-D: Diarrhea-predominant irritable bowel syndrome
IBS-SI: IBS severity index
LEfSe: Linear discriminant analysis effect size
NDG: Near Donor Group
PCoA: Principal coordinate analysis
QOL: Quality of life
