# Supplementary material for: Fecal microbiota transplantation in the treatment of irritable bowel syndrome: a single-center prospective study in Japan
Source: BMC Gastroenterol. 2022 Jul 14;22:342. doi: 10.1186/s12876-022-02408-5 (PMC9284895; doi:10.1186/s12876-022-02408-5)
Supplement: Supplementary file 1 — Additional file 1: Table S1. Screening Test and Interview of Donors. [file 12876_2022_2408_MOESM1_ESM.docx]

**Supplementary Table 1. Screening Test and Interview of Donors**

| Stool test  Fecal culture, CD toxin, insect eggs, fecal occult blood |
| --- |
| Blood collection  Blood count, biochemistry, HBs-Ag, HCV-Ab, syphilis, HIV, T-SPOT |
| Medical interview |
| - Do you have HIV or Hepatitis B? - Are there any banned substances in use? - Do you have a history of blood transfusions? - Have you had any significant trauma in the past 6 months? - Is it the case that you have you not seen a medical care provider in the past 6 months? - Have you traveled abroad in the past 6 months? - Have you had any vaccinations in the past 6 months? - Are you a medical professional? - Is it the case that you do not work with animals? - Do you have any symptoms of long-term abnormal stools, such as irritable bowel syndrome, chronic constipation, or chronic diarrhea? - Do you have a collagen disease? - Do you have a family history of cancer? - Do you have a history of any diseases that you are concerned about? - Do you have a history of mental illness? - Is your BMI less than 25? - Is it the case that you have not taken any regular medications in the last 6 months or more? |

CD: *Clostridium difficile*; HBs-Ag: Hepatitis B surface antigen; HCV-Ab: Hepatitis C virus antibody; HIV: human immunodeficiency virus; T-SPOT: tuberculosis test; BMI; body mass index.
