# Supplementary figures and images for: Fecal microbiota transplantation in the treatment of irritable bowel syndrome: a single-center prospective study in Japan
Source: BMC Gastroenterol. 2022 Jul 14;22:342. doi: 10.1186/s12876-022-02408-5 (PMC9284895; doi:10.1186/s12876-022-02408-5)

## Slide 1
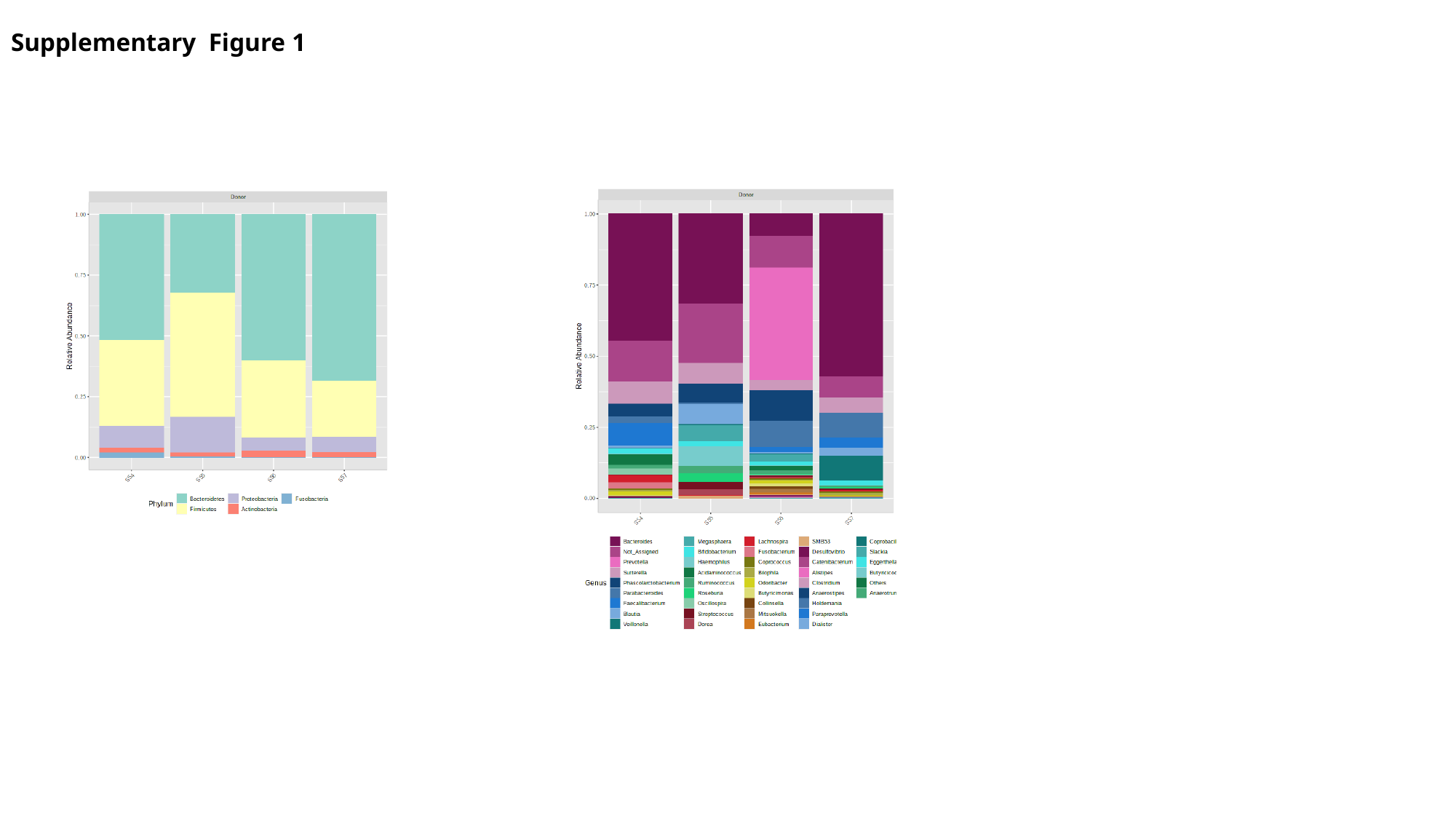

Supplementary Figure 1

Supplement: Supplementary file 2 — Additional file 2: Fig. S1. The relative abundance of all donors’ microbiome at the phylum and genus level. [file 12876_2022_2408_MOESM2_ESM.pptx]

## Slide 1
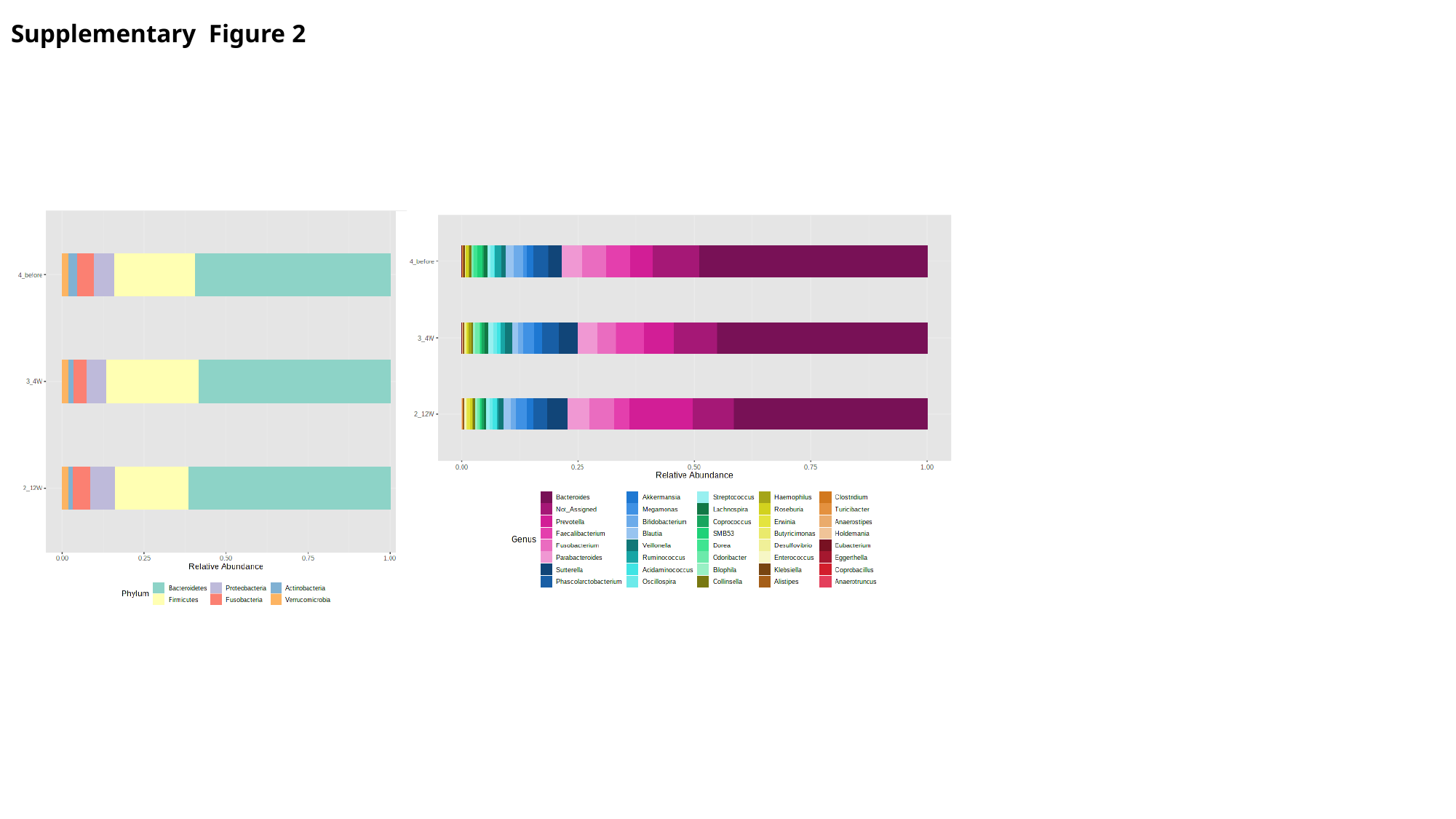

Supplementary Figure 2

Supplement: Supplementary file 3 — Additional file 3: Fig. S2. The relative abundance of all patients’ microbiome at the phylum and genus level. [file 12876_2022_2408_MOESM3_ESM.pptx]

## Slide 1
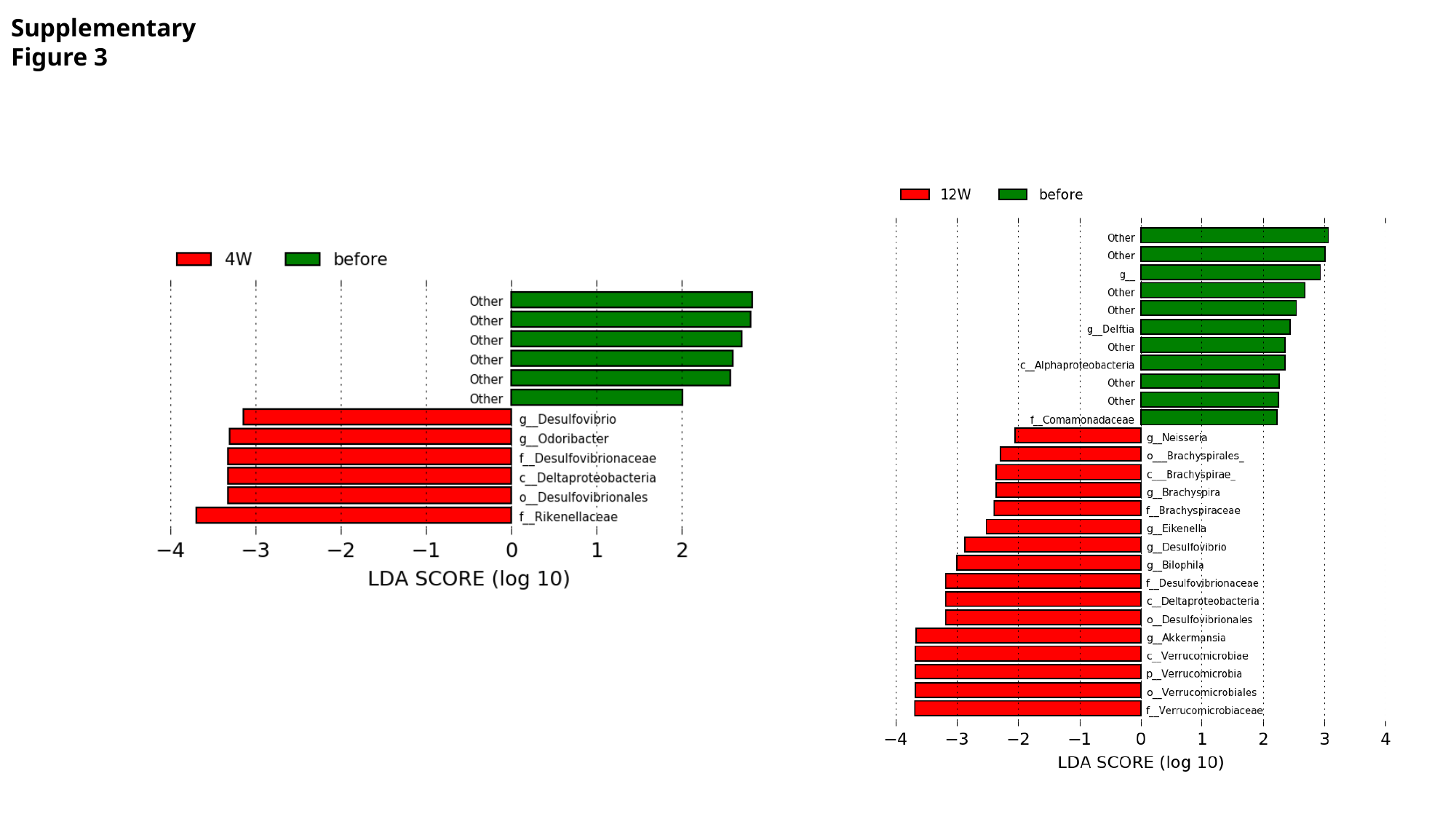

Supplementary Figure 3

Supplement: Supplementary file 4 — Additional file 4: Fig. S3. Differences in all patients’ microbiome before and after Fecal Microbiota Transplantation. [file 12876_2022_2408_MOESM4_ESM.pptx]

## Slide 1
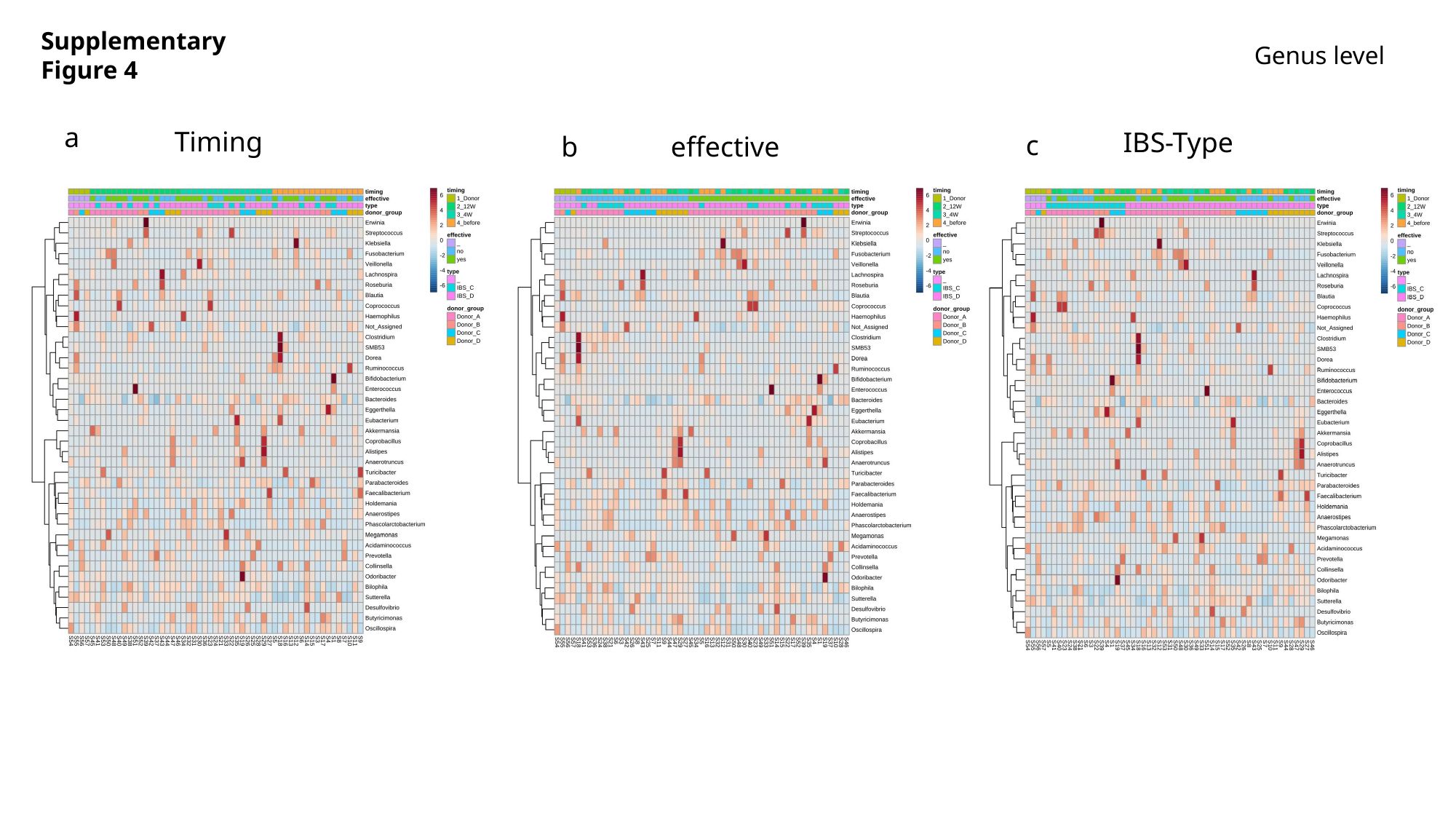

Supplementary
Figure 4
	Genus level
a
	Timing
	IBS-Type
c
b
	effective

Supplement: Supplementary file 5 — Additional file 5: Fig. S4. Each heat map was sorted by timing, effectivity, and IBS-type. [file 12876_2022_2408_MOESM5_ESM.pptx]
